# Ovariectomy increases the incidence and diameter of abdominal aortic aneurysm in a hypoperfusion-induced abdominal aortic aneurysm animal model

**DOI:** 10.1038/s41598-019-54829-0

**Published:** 2019-12-04

**Authors:** Chie Miyamoto, Hirona Kugo, Keisuke Hashimoto, Tatsuya Moriyama, Nobuhiro Zaima

**Affiliations:** 1 Department of Applied Biological Chemistry, Graduate School of Agriculture, Kindai University, 204-3327 Nakamachi, Nara City, Nara, 631-8505 Japan; 2 Agricultural Technology and Innovation Research Institute, Kindai University, Nara, Japan

**Keywords:** Aneurysm, Chronic inflammation, Risk factors

## Abstract

Abdominal aortic aneurysm (AAA) is a vascular disease characterized by weakening of the vascular walls. Male sex is a risk factor for AAA, and peak AAA incidence occurs in men 10 years earlier than in women. However, the growth rate of AAA is faster in women, and women have a higher mortality due to AAA rupture. The mechanisms underlying sex-related differences in AAA remain unknown. Herein, we evaluated the effects of ovariectomy (OVX) on AAA in rats. Upon evaluation of the effects of OVX and AAA induction, AAA incidence rate and the aneurysm diameter increased in the OVX group. However, the histopathology in the developed AAA wall was not different between groups. When the effects of OVX on the vascular wall without AAA induction were evaluated, elastin and collagen levels were significantly decreased. Furthermore, the level of matrix metalloproteinase-9 significantly increased in the OVX group. According to our results, it is speculated that decreased levels of collagen and elastin fibers induced by OVX might be involved in increased incidence rate and diameter of AAA. Weakening of the vascular wall before the onset of AAA might be one reason for the faster rate of AAA growth in women.

## Introduction

Abdominal aortic aneurysm (AAA) is a vascular disease involving progressive dilation of the abdominal aorta. In light of the very high mortality rate due to AAA rupture, its prevention is an issue of great importance. However, therapeutic drugs to prevent the progression and rupture of AAA have not been established^1^. Previous studies have reported that AAA is closely associated with weakening of the vascular wall induced by inflammation^2^. Immune cells such as macrophages and monocytes release proteases and inflammatory cytokines, including matrix metalloproteinases (MMPs) and monocyte chemoattractant protein-1 (MCP-1), which degrade the elastin and collagen fibers in the vascular wall^3^. Degradation of the arterial wall accompanied by high blood pressure is a predisposing factor for the development of AAA. It has been reported that vascular wall hypoperfusion induced by stenosis of the vasa vasorum leads to the development of AAA^4,5^.

Male sex is a non-modifiable risk factor for AAA^6,7^. Previous studies indicated that AAA occurred 4 times as often in men as in women^8^. In the UK, a current AAA screening program is considered to be cost-effective for men but not for women^9^. However, sex -based differences in AAA should be taken into account to understand the characteristic pathology of AAA in women. Women tend to be diagnosed with AAA 10 years later than men^10^. The risk of AAA rupture was 3–4 times higher in women than in men, and the aneurysm growth rate was higher in women than in men^11,12^. Women were at a higher risk for 30-day mortality and major complications following endovascular aneurysm repair and open AAA repair^13^. Most women with AAA are post-menopausal and women with larger AAA were found to experience earlier menopause^14,15^. The hypoactivity of ovary in menopause causes the decreased level of estrogens which are associated with oxidative stress and inflammation in the vascular wall^16^. These reports suggest that hypoactivity of the ovary is involved in the characteristic AAA events in women. However, the precise mechanisms underlying characteristic AAA events in women remain unknown. In this study, we evaluated the effects of ovariectomy (OVX) on AAA and show that OVX increases the abdominal aortic aneurysm incidence rate and diameter and causes decreased level of collagen and elastin fibers.

## Results

### OVX increases the incidence rate AAA and aortic diameter

**Figure 1:**
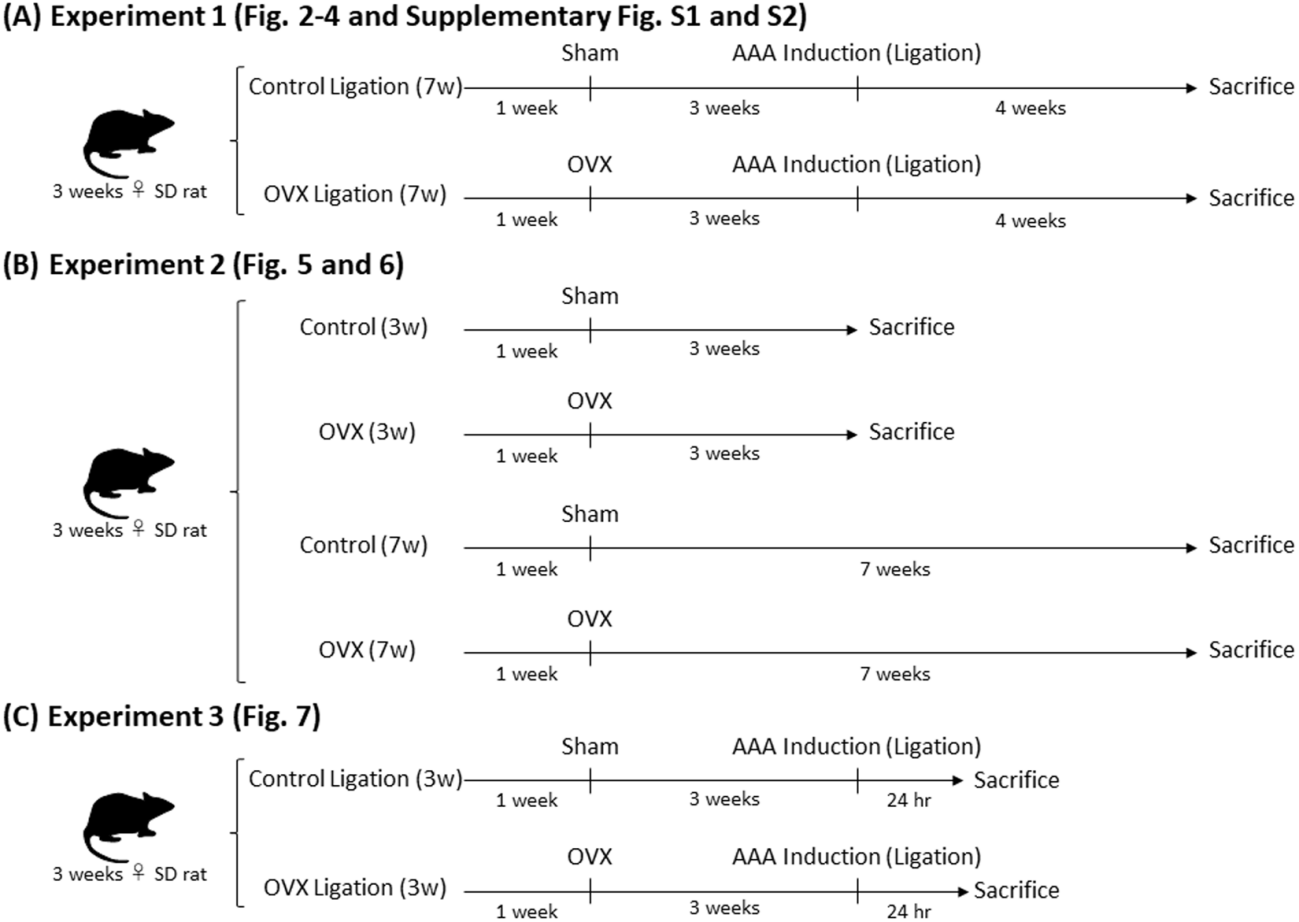
Experimental schema. (**A**) Schema of the experiment 1 protocol. (**B**) Schema of the experiment 2 protocol. (**C**) Schema of the experiment 3 protocol.

In experiment 1 (Fig. 1A), initial body weight (g), final body weight (g), and food intake (kcal/day) in the hypoperfusion-induced AAA model rats did not differ significantly between the control ligation group and the OVX ligation group (Supplementary Table S1). Oviduct weight (g/100 g body weight) and serum 17β-estradiol were significantly lower in the OVX ligation group than in the control ligation group, indicating successful treatment of OVX (Supplementary Table S1).

**Figure 2:**
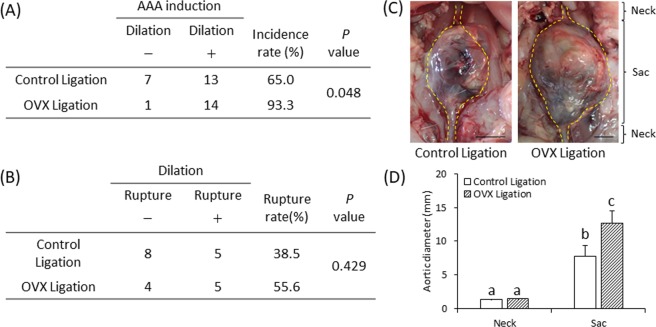
Effects of OVX on abdominal aortic aneurysm (AAA) incident, rupture, and aortic diameter. The effect of OVX on AAA incidence rate (**A**) and AAA rupture rate (**B**). Data are presented as number of rats. P values of the Fisher’s exact test. AAA formation was defined as dilation ratio > 2.0. The dilation rate = maximal aneurysm diameter (sac) / non-dilated vascular diameter (neck). (**C**) Representative images of the abdominal aorta from AAA induction in the control ligation and the OVX ligation groups (scale bar = 5 mm). (**D**) Quantitative analysis of aortic diameter. Data are represented as mean ± S.E.M. (**A**) Control ligation group (n = 20), OVX ligation group (n = 14). (**B**) Control ligation group (n = 13), OVX ligation group (n = 9). (**D**) Control ligation group (n = 13), OVX ligation group (n = 14). Values with different letters are significantly different (P < 0.05).

The AAA incidence rates of the control ligation group and OVX ligation group are shown in Fig. 2A. The increase in the AAA incidence rate was more in the OVX ligation group than in the control ligation group (Fig. 2A). AAA rupture rates were not significantly different between the control ligation group and the OVX ligation group (Fig. 2B). An image of a formed AAA is shown in Fig. 2C. We observed an area with a non-dilated diameter (neck) and an area with a dilated aortic diameter (sac) in both groups (Fig. 2C). The neck diameter did not differ significantly between the control ligation group and the OVX ligation group (Fig. 2D). The sac was significantly larger than the neck in each group (Fig. 2D). Sac diameter was significantly larger in the OVX ligation group than in the control ligation group (Fig. 2D).

### OVX does not alter adventitial ectopic adipocytes and fibers degradation in the developed AAA wall

Since a previous study reported that the abnormal appearance of adipocytes was involved in AAA in human and animal models^17–20^, we evaluated adipocytes in the adventitia. They were observed in both the control ligation and OVX ligation groups after AAA induction (Supplementary Fig. S1A-D). These areas were stained positively by Oil red O staining (Supplemental Figs. S1E–H). The number and size (μm^2^/cell) of adipocytes in the vascular wall did not differ significantly between the control ligation and OVX ligation groups (Supplementary Figs. S1I and 1J).

**Figure 3:**
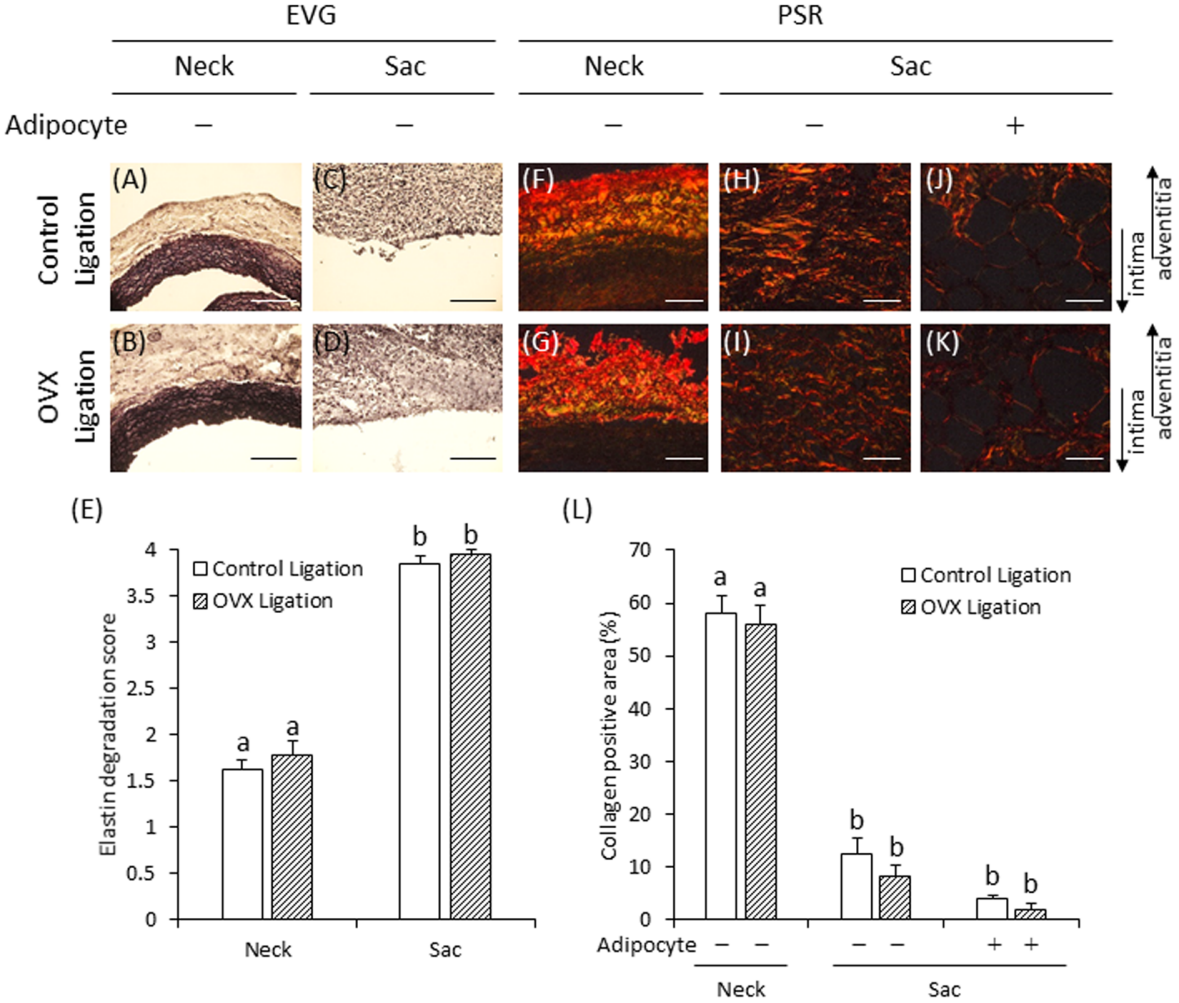
Elastica van Gieson (EVG) and Picrosirius Red (PSR) staining of the AAA wall in experiment 1 (**A–D**) Representative images of EVG staining (scale bar = 100 µm). (**E**) Elastin degradation scores in the control ligation and OVX ligation groups. (**F–K**) Representative images of PSR staining (scale bar = 50 µm). (**L**) Quantification of the collagen-positive area of the vascular wall in the control ligation and OVX ligation groups. Data are represented as mean ± S.E.M. (**E,L**) Control ligation group (n = 13), OVX ligation group (n = 9). Values with different letters are significantly different (*P* < 0.05). The arrows show the direction of intima and adventitia. In the case of enlarged figure, all layers of arterial wall were not shown.

Representative pictures of elastic fibers were shown (Fig. 3A–D). The elastin degradation score in the AAA sac was significantly higher than that in the AAA neck in both the control and OVX ligation groups; however, there was no significant difference between the control and OVX ligation groups (Fig. 3E). Representative pictures of collagen fibers are shown (Fig. 3F–K). The collagen-positive area in the AAA sac was significantly smaller than that in the AAA neck in both the control and OVX ligation groups. The collagen-positive area around the adipocytes in the AAA sac tended to show a greater decrease than that shown by the area without adipocytes. The collagen-positive areas were not significantly different between the control and OVX ligation groups (Fig. 3L).

### OVX does not alter MMP and monocyte/macrophage expression in the developed AAA vascular wall

**Figure 4:**
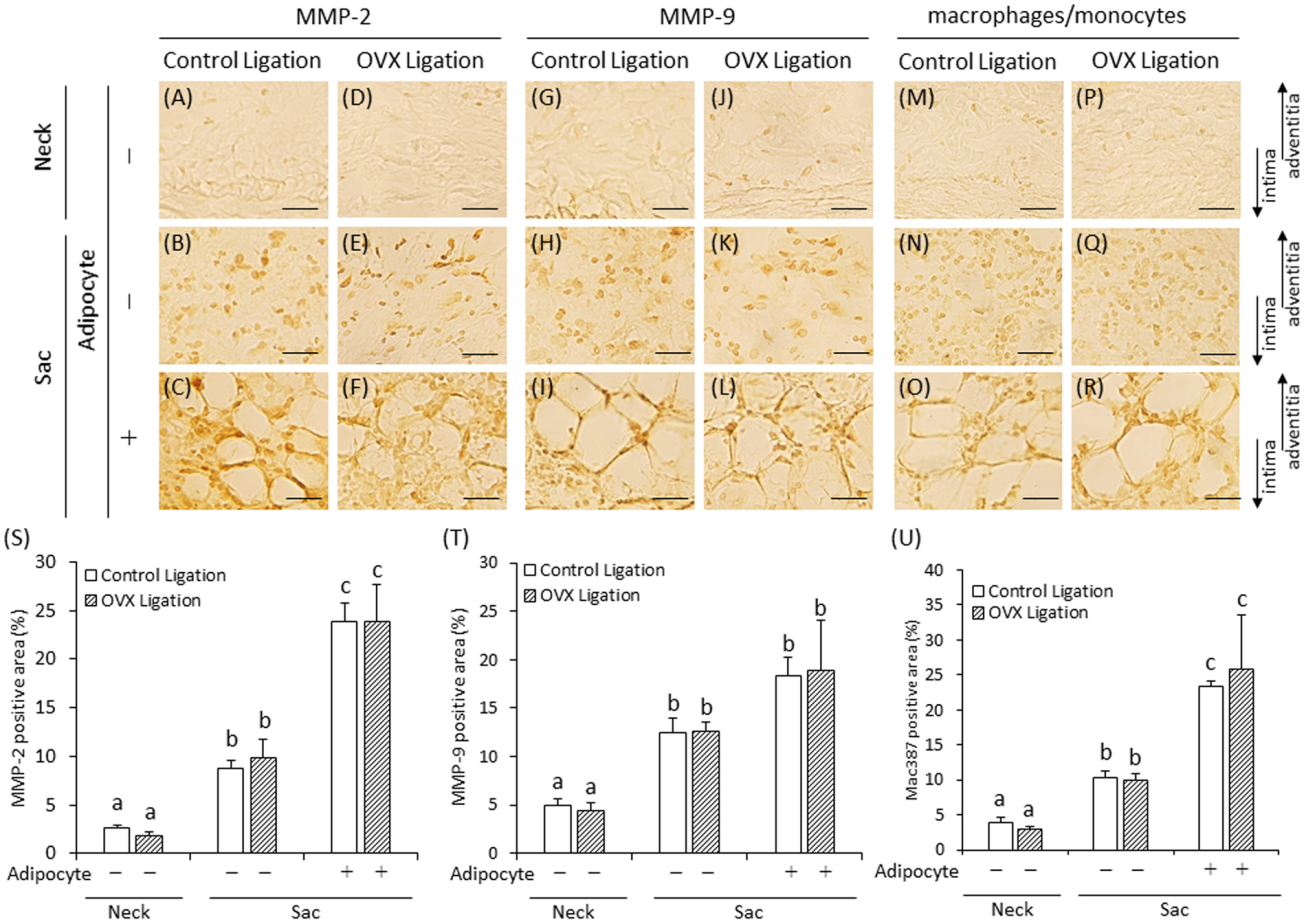
Immunohistochemical staining for matrix metalloproteinases (MMPs) and macrophage/monocyte of AAA wall in experiment 1. Abdominal aortic aneurysm (AAA) sac areas from the two experimental groups were divided into those without adipocytes (−) (**B,E,H,K,N,Q**) and those with adipocytes ( + ) (**C,F,I,L,O,R**). (**A–F**) Representative images of immunostaining for MMP-2 (scale bar = 30 µm). (**G–L**) Representative images of immunostaining for MMP-9 (scale bar = 30 µm). (**M–R**) Representative images of immunostaining for macrophage/monocyte (scale bar = 30 µm). (**S**) Quantification of MMP-2 positive areas of the vascular wall. (**T**) Quantification of MMP-9 positive areas of the vascular wall. (**U**) Quantification of macrophage/monocyte positive areas of the vascular wall. (**S,T**) Control ligation group (n = 12), OVX ligation group (n = 8). (**T**) Control ligation group (n = 11), OVX ligation group (n = 8). Values with different letters are significantly different (P < 0.05). The arrows show the direction of intima and adventitia. In the case of enlarged figure, all layers of arterial wall were not shown.

The expression of MMP-2, MMP-9, and monocytes/macrophages is shown in Fig. 4A–R. Negative controls are shown in supplementary Fig. S2. The positive areas for MMP-2, MMP-9, and monocytes/macrophages in the AAA sac were significantly more than for the positive areas in the AAA neck (Fig. 4S–U). However, these positive areas in the control ligation group were not significantly different from those in the OVX ligation group (Fig. 4S–U). Immunohistochemical staining for MMP-12 and MCP-1 is shown in Supplementary Fig. S3A–L. The areas positive for MMP-12 and MCP-1 were not significantly different between the control ligation group and the OVX ligation group (Supplementary Figs. S3M and 2N).

### OVX degrades elastin and collagen fibers in the vascular wall

Given the lack of significant differences between the control ligation and OVX ligation groups, we evaluated the effects of OVX on the vascular wall without AAA induction in experiment 2 (Fig. 1B). At 3 weeks after OVX, initial body weight (g), final body weight (g), and food intake (kcal/day) did not differ significantly between the control group and the OVX group (Supplementary Table S2). Moreover, oviduct weight (g/100 g body weight) was significantly lower in the OVX group than in the control group (Supplementary Table S2). At 7 weeks after OVX, the final body weight (g) and food intake (kcal/day) were significantly higher in the OVX group than in the control group (Supplementary Table S2). Furthermore, oviduct weight (g/100 g body weight) and serum 17β-estradiol were significantly lower in the OVX group than in the control group (Supplementary Table S2).

**Figure 5:**
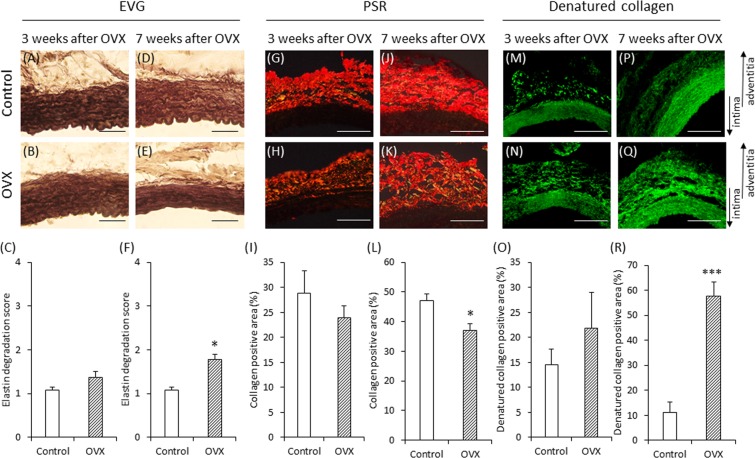
Elastica van Gieson (EVG), Picrosirius Red (PSR) and denatured collagen staining of vascular wall in experiment 2 (**A,B,D,E**) Representative images of EVG staining (scale bar = 50 µm). (**G,H,J,K**) Representative images of PSR staining (scale bar = 100 µm). (**M,N,P,Q**) Representative images of denatured collagen staining (scale bar = 100 µm). Elastin degradation scores in the control and OVX groups on 3 weeks after OVX (**C**) and on 7 weeks after OVX (**F**). Quantification of the collagen-positive area of the vascular wall in the control and OVX groups on 3 weeks after OVX (**I**) and on 7 weeks after OVX (**L**). Quantification of the denatured collagen positive area of the vascular wall in the control and OVX groups on 3 weeks after OVX (**O**) and on 7 weeks after OVX (**R**). (**C,F,I**) Control group (n = 8), OVX group (n = 8). (**L,O**) Control group (n = 7), OVX group (n = 9). (**R**) Control group (n = 7), OVX group (n = 8). *P < 0.05, ***P < 0.001. The arrows show the direction of intima and adventitia.

The elastin degradation score did not differ significantly between the OVX group and the control group at 3 weeks after OVX (Fig. 5A–C); however, the increase in the OVX group was significantly more than that in the control group at 7 weeks after OVX (Fig. 5D–F). Although the collagen-positive area did not differ significantly between the control group and the OVX group at 3 weeks after OVX (Fig. 5G–I), the decrease in the OVX group was significantly more than that in the control group at 7 weeks (Fig. 5J–L). The denatured collagen-positive area did not differ significantly between groups at 3 weeks after OVX (Fig. 5M–O); however, the increase was significantly more in the OVX group than in the control group at 7 weeks (Fig. 5P–R).

### OVX increases the expression of MMP-9 but not MMP-2

**Figure 6:**
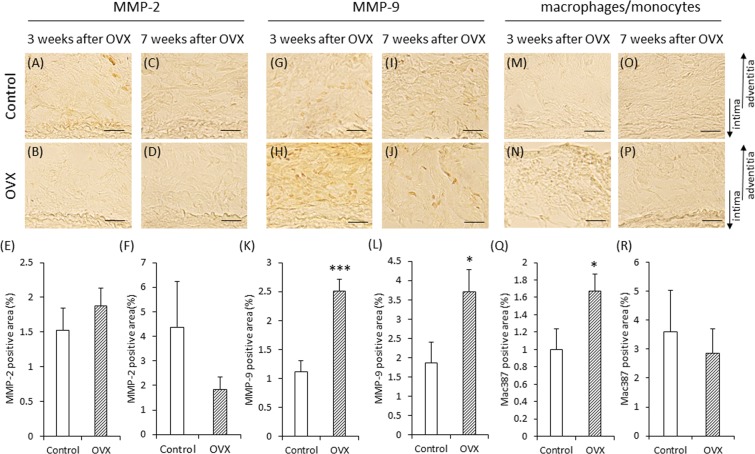
Immunohistochemical staining for matrix metalloproteinases (MMPs) and macrophage/monocyte of vascular wall in experiment 2. (**A,B,D,E**) Representative images of immunostaining for MMP-2 (scale bar = 30 µm). (**G,H,J,K**) Representative images of immunostaining for MMP-9 (scale bar = 30 µm). (**M,N,P,Q**) Representative images of immunostaining for macrophage/monocyte (scale bar = 30 µm). Quantification of MMP-2 positive areas of the vascular wall on 3 weeks after OVX (**C**) and on 7 weeks after OVX (**F**). Quantification of MMP-9 positive areas of the vascular wall on 3 weeks after OVX (**I**) and on 7 weeks after OVX (**L**). Quantification of macrophage/monocyte positive areas of the vascular wall on 3 weeks after OVX (**O**) and on 7 weeks after OVX (**R**). (**E,K**) Control group (n = 8), OVX group (n = 8). (**F**) Control group (n = 6), OVX group (n = 8). (**L,Q**) Control group (n = 7), OVX group (n = 9). (**R**) Control group (n = 7), OVX group (n = 8). *P < 0.05, ***P < 0,001. The arrows show the direction of intima and adventitia. In the case of enlarged figure, all layers of arterial wall were not shown.

Immunohistochemical staining showed the expression of MMP-2, MMP-9, and monocytes/macrophages in each group (Fig. 6A–D,G–J,M–P). The area positive for MMP-2 did not differ significantly between the control group and the OVX group at either 3 or 7 weeks after OVX (Fig. 6E,F). The increase in the area positive for MMP-9 was significantly more in the OVX group than in the control group at both 3 and 7 weeks after OVX (Fig. 6K,L). The increase in the area positive for monocytes/macrophages was significantly more in the OVX group than in the control group at 3 weeks after OVX (Fig. 6Q), with no significant intergroup difference at 7 weeks after OVX (Fig. 6R).

### OVX does not alter acute inflammation induced by AAA induction

**Figure 7:**
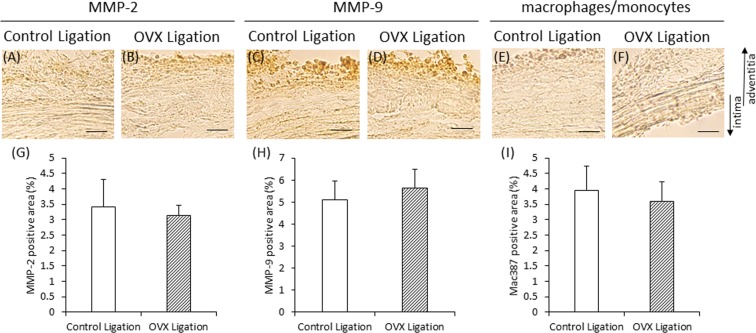
Immunohistochemical staining for matrix metalloproteinases (MMPs) and macrophage/monocyte of vascular wall in experiment 3. (**A,B**) Representative images of immunostaining for MMP-2 (scale bar = 30 µm). (**C,D**) Representative images of immunostaining for MMP-9 (scale bar = 30 µm). (**E,F**) Representative images of immunostaining for macrophage/monocyte (scale bar = 30 µm). (**G**) Quantification of MMP-2 positive areas of the vascular wall. (**H**) Quantification of MMP-9 positive areas of the vascular wall. (**I**) Quantification of macrophage/monocyte positive areas of the vascular wall. (**G,H**) Control ligation group (n = 8), OVX ligation group (n = 8). (**I**) Control ligation group (n = 8), OVX ligation group (n = 9). The arrows show the direction of intima and adventitia. In the case of enlarged figure, all layers of arterial wall were not shown.

In experiment 3, we estimated MMPs and monocytes/macrophages in the vascular wall at 24 hours after AAA induction in order to evaluate the effects of OVX on acute inflammation caused by AAA induction (Fig. 1C). Representative pictures of MMP-2, MMP-9, and monocytes/macrophages are shown in Fig. 7A–F. The areas positive for MMP-2, MMP-9, and monocytes/macrophages in control ligation group were not significantly different from those in the OVX ligation groups (Fig. 7G–I).

## Discussion

In this study, we evaluated the effects of OVX on AAA and observed significantly greater increases in the incidence rate and diameter of AAA in the OVX ligation group than in the control ligation group. Thus far, most females with AAA in clinical studies have been postmenopausal, which suggests that hypoactivity of ovary may be a predisposing factor for AAA in women^15^. In the present study, we performed a histological analysis of AAA to investigate the mechanisms underlying the increased AAA incidence rate in the OVX ligation group, and no significant difference in pathologies was observed between the control ligation and OVX ligation groups. Then, we evaluated the effects of OVX on the vascular wall without AAA induction. Vascular fibers, such as elastin and collagen, were significantly degraded in the OVX group at 7 weeks after OVX, in line with the finding of Johnston *et al*. that the staining of smooth muscle cell markers and elastin was decreased in OVX mice^21^. These results suggest that OVX in itself does not cause the formation of AAA but causes weakening of the vascular wall, which is implicated in the increased incidence of AAA and in larger-sized AAA after the induction of hypoperfusion in the vascular wall.

The increase in the area positive for MMP-9 was significantly more in the OVX group than in the control group at both 3 and 7 weeks after OVX. In contrast, the area positive for MMP-2 did not differ between the control group and the OVX group. The increase in the area positive for macrophage/monocyte expression was significantly more in the OVX group than in the control group at 3 weeks after OVX. These results suggest that OVX is involved in the increased level of MMP-9, but not of MMP-2. However, the increased level of MMP-9 can only be partly attributed to the increased infiltration of macrophages/monocytes, and further studies are needed. At 24 hours after AAA induction, the areas positive for MMPs and macrophages/monocytes did not differ between the control ligation group and the OVX ligation group; therefore, we speculate that OVX did not lead to hypoperfusion-induced exacerbation of vascular inflammation in the vascular wall. Because human AAA is associated with many complex factors, further studies are needed to clarify the effect of OVX on AAA.

A previous study using an elastase aortic perfusion model showed that AAAs in female mice were smaller than those in male mice^21,22^. However, the size of AAAs formed in OVX female mice was equivalent to that in male mice^21^. Furthermore, AAA size in female mice with genetic deletion of aromatase, an estrogen synthesis enzyme, reached that observed in male mice^21^. A previous study showed that 17β-estradiol administration blunted AAA progression-induced angiotensin II in OVX female mice^15^. Taken together, these results suggest that AAA development in the context of OVX may be promoted by decreased levels of female sex hormones. In this study, we observed a decreased level of 17β-estradiol in the OVX group. The decreased level of 17β-estradiol can be involved in the increased level of MMP-9. 17β-estradiol reportedly inhibits NFκB-dependent inflammation in rat aortic smooth muscle cells via estrogen receptor-β^23^. Xing *et al*. reported that 17β-estradiol modulate NFκB signaling by two distinct mechanisms: promotion of new IκBα synthesis, which is associated with negative feedback in NFκB signaling, and direct inhibition of biding of NFκB to the promoters of inflammatory genes. A functional NFκB site occurs in the MMP-9 promoter^24,25^ and NFκB is required for upregulation of MMP-9 but not MMP-2^26^.

In general, estrogen is known to play a role in vascular wall protection in premenopausal women. It was reported that women with large AAAs reached menopause at an earlier age, and estrogen is believed to exert protective effects on AAA development in women^14^. One previous report showed that hormone replacement therapy (HRT) reduced the AAA incidence rate,^27^ whereas another report showed that HRT increased the incidence of AAA,^28^ revealing a lack of consensus in terms of the effects of HRT on AAA. According to the timing hypothesis, the effects of hormone therapy on cardiovascular disease depend on the timing of the initiation of hormone therapy relative to menopause^29,30^. Our results showed that OVX causes decrease in 17β-estradiol and gradual degradation of fibers in the vascular wall; therefore, our results were not inconsistent with the timing hypothesis regarding AAA in women. However, it remains inconclusive whether hormone therapy for AAA is beneficial in postmenopausal women.

Abnormal appearance of adipocytes in the AAA wall is reportedly associated with AAA development or rupture both in the human AAA and animal AAA models. In the AAA model animal, mesenchymal stem cells in perivascular adipose tissue were abnormally differentiated into adipocytes under conditions of hypoperfusion in the vascular wall^31^. Adipocytes can exacerbate the degradation of collagen fibers in the AAA wall^17,18,32^. Abnormal appearance of adipocytes was also observed in human AAA,^17,19,33,34^ but not in human popliteal artery aneurysms, which have an extremely low propensity to rupture^19^. Biomechanical analysis shows that the adipocytes in the AAA wall are reportedly associated with weakness of human AAA wall^20^. In this study, adipocyte number and area were not different between the control ligation and OVX ligation groups, suggesting OVX-induced increase of AAA incidence rate and diameter are not directly involved in adipocytes in AAA wall. This might be because of the different stages of AAA pathology. Our results showed that OVX degrade fibers before AAA induction, while adipocytes reportedly appeared two weeks after the induction in previous study^31^.

In conclusion, our results suggest that OVX increased the incidence rate and diameter of AAA, which might be attributed to the decreased levels of collagen and elastin fibers and the increased level of MMP-9. Lower levels of these fibers before AAA onset may contribute to the faster rate of AAA growth in women. The limitation of this study is the lack of data of human pathology owing to the difficulty of comparison of vascular wall in similar condition to this study. In addition, we did not show the effect of exogenous sex hormone administration on OVX treated groups. The limitation of the AAA model used in this study is the lack of atherosclerotic lesion in AAA wall. Further studies are needed to complement this study.

## Methods

### Animals

All animal experiments were approved by the Kindai University Animal Care and Use Committee and performed according to the Kindai University Animal Experimentation Regulations (approval number: KAAG-25–001). Three-week-old female Sprague-Dawley rats were obtained from SHIMIZU Laboratory Supplies Co., Ltd. (Kyoto, Japan) They were provided with food and tap water ad libitum in a humidity-controlled room with a 12-hour light/dark cycle. Diet composition is shown in Supplementary Table S3. Room temperature was maintained at 25 ± 1 °C. We performed the following 3 experiments (Fig. 1A–C).

Experiment 1 (Fig. 1A): The rats were divided into the control ligation group (sham; surgery without ovary removal + AAA induction) and the OVX ligation group (surgery with ovary removal + AAA induction). After a 1-week habituation period, the control ligation group and the OVX ligation group underwent either a sham operation or OVX. At 3 weeks after the sham operation or OVX, the abdominal aorta was ligated over an inserted catheter in all rats to induce an AAA. At 4 weeks after AAA induction, the aortic diameters were measured, and the rats were sacrificed.

Experiment 2 (Fig. 1B): The rats were divided into the control group (sham; surgery without ovary removal) and the OVX group (surgery with ovary removal). After a 1-week habituation period, the control group and the OVX group underwent either a sham operation or OVX. At 3 or 7 weeks after the sham operation or OVX, the aortic diameters were measured, and the rats were sacrificed.

Experiment 3 (Fig. 1C): The rats were divided into the control ligation group and the OVX ligation group, as in experiment 1. After a 1-week habituation period, the control ligation group and the OVX ligation group underwent either a sham operation or OVX. At 3 weeks after the sham operation or OVX, the abdominal aorta was ligated over an inserted catheter in all rats to induce vascular hypoperfusion. At 24 hours after AAA induction, the diameters of aortae were measured, and animals were sacrificed.

When a rat died of AAA rupture, the aortic diameter was measured, and the abdominal aorta was immediately isolated. AAA was identified when the following features were observed: abdominal aortic dilation, presence of a blood clot or hemorrhage around the abdominal aorta (in cases of rupture), and destruction of collagen and elastic fibers in the vascular wall. In experiment 1, 5 rats in the OVX ligation group were excluded from the vascular wall analysis because they died due to causes other than rupture. All surgeries were performed under anesthesia using medetomidine, midazolam, and butorphanol. All efforts were made to minimize suffering.

### Induction of hypoperfusion of the abdominal aortic wall

Hypoperfusion of the abdominal aortic wall was induced as previously described^5,17^. First, the infra-renal aorta was exfoliated from the perivascular tissue. Vessels branching from the abdominal aorta were ligated with a 5–0 silk string (Akiyama-seisakusyo, Tokyo, Japan) to block the blood supply. A plastic catheter (Medikit, Tokyo, Japan) shortened to 9 mm in length was inserted via a small incision adjacent to the renal artery branches, and the incision was repaired with a 6–0 monofilament string (Alfresa Pharma, Osaka, Japan). The abdominal aorta was ligated with a 5–0 silk string and a plastic catheter. The diameter of the abdominal aorta was measured using digital calipers (A&D, Tokyo, Japan). Isolated tissues were fixed in 4% paraformaldehyde (Nacalai Tesque, Kyoto, Japan), soaked in sucrose (10%, 15%, and 20%), embedded in O.C.T. Compound (Sakura Finetek Japan Co., Ltd., Tokyo, Japan), and stored at −80 °C until use.

### Histological analysis

Isolated aorta cross-sections (10-µm-thick) were prepared using a cryostat (CM1850; Leica Microsystems, Wetzlar, Germany) and mounted on glass slides. The denatured collagen was measured by using collagen hybridizing peptide (3Helix, Utah, USA) Quantitative analysis of the histological staining was performed using ImageJ software (National Institutes of Health, Bethesda, MD, USA). Areas within 100 µm of an adipocyte were defined as “areas with adipocytes.” Elastic fibers were categorized into 4 grades: grade 1, intact elastic fibers; grade 2, lack of wave form; grade 3, thinning of the wave form and/or partial disappearance of elastic fibers; and grade 4, complete disappearance of elastic fibers^17,35^. Immunohistochemical staining were performed as previously described^35^.

### Serum 17β-estradiol assay

Serum 17β-estradiol was quantified using the 17β-estradiol ELISA Kit (Tokiwa Chemical Industries, Tokyo, Japan).

### Statistical analysis

Values are expressed as mean ± SEM. The Chi-square test, Fisher’s exact test (for situations with small frequencies), or Mann Whitney U test were used for categorical variables. Statistical differences were determined using a two-sided Student’s *t*-test. Multiple comparisons between groups were performed using the Tukey-Kramer test. The statistical difference in scoring data was determined by Scheffe’s test. Differences of P < 0.05 were considered significant. StatView 5.0 software (SAS Institute, Cary, NC, USA) were used for statistical analyses.

## Supplementary information

Supplementary information
