# Supplementary material for: Ovariectomy increases the incidence and diameter of abdominal aortic aneurysm in a hypoperfusion-induced abdominal aortic aneurysm animal model
Source: Sci Rep. 2019 Dec 4;9:18330. doi: 10.1038/s41598-019-54829-0 (PMC6892790; doi:10.1038/s41598-019-54829-0)
Supplement: Supplementary file 1 — Supplementary information [file 41598_2019_54829_MOESM1_ESM.pdf]

Supplementary Information

**Title; Ovariectomy increases the incidence and diameter of abdominal aortic aneurysm in a hypoperfusion-induced abdominal aortic aneurysm animal model**

Chie Miyamoto[1], Hirona Kugo[1], Keisuke Hashimoto[1], Tatsuya Moriyama[1,2], Nobuhiro Zaima[1,2*]

[1] Department of Applied Biological Chemistry, Graduate School of Agriculture, Kindai University, 204-3327 Nakamachi, Nara City, Nara 631-8505, Japan

[2] Agricultural Technology and Innovation Research Institute, Kindai University, Nara, Japan

*C.M. and H.K. contributed equally to this work.

**Supplementary Figure S1** Quantification of adipocytes.

(A-D) Representative images of hematoxylin-eosin-stained samples (scale bar = 200 μm). (E-H) Representative images of Oil Red O staining (scale bar = 200 μm). (I) Quantification of number of adipocytes. (J) Quantification of adipocyte area. Control group (n = 13), high-fat group (n = 9). The arrows show the direction of intima and adventitia. In the case of enlarged figure, all layers of arterial wall were not shown.

**Supplementary Figure S2 Negative controls of immunohistochemical staining**

Representative images of negative stain controls of MMP-2 (A and B), MMP-9 (C and D), and macrophage/monocyte (E and F). Scale bar = 30 μm.

**Supplementary Figure S3** Immunohistochemical staining for MMP-12 and MCP-1 of AAA wall in experiment 1.

Abdominal aortic aneurysm (AAA) sac areas from the two experimental groups were divided into those without adipocytes (-) (B, E, H and K) and those with adipocytes (+) (C, F, I and L). (A-F) Representative images of immunostaining for MMP-12 (scale bar = 30 μm). (G-L) Representative images of immunostaining MCP-1(scale bar = 30 μm). (M) Quantification of MMP-12 positive areas of the vascular wall. (N) Quantification of MCP-e1-positive areas of the vascular wall. (M) Control ligation group (n = 12), OVX ligation group (n = 8). (N) Control ligation group (n = 11), OVX ligation group (n = 8). Values with different letters are significantly different ($P < 0.05$). The arrows show the direction of intima and adventitia. In the case of enlarged figure, all layers of arterial wall were not shown.

Supplementary Figure S1

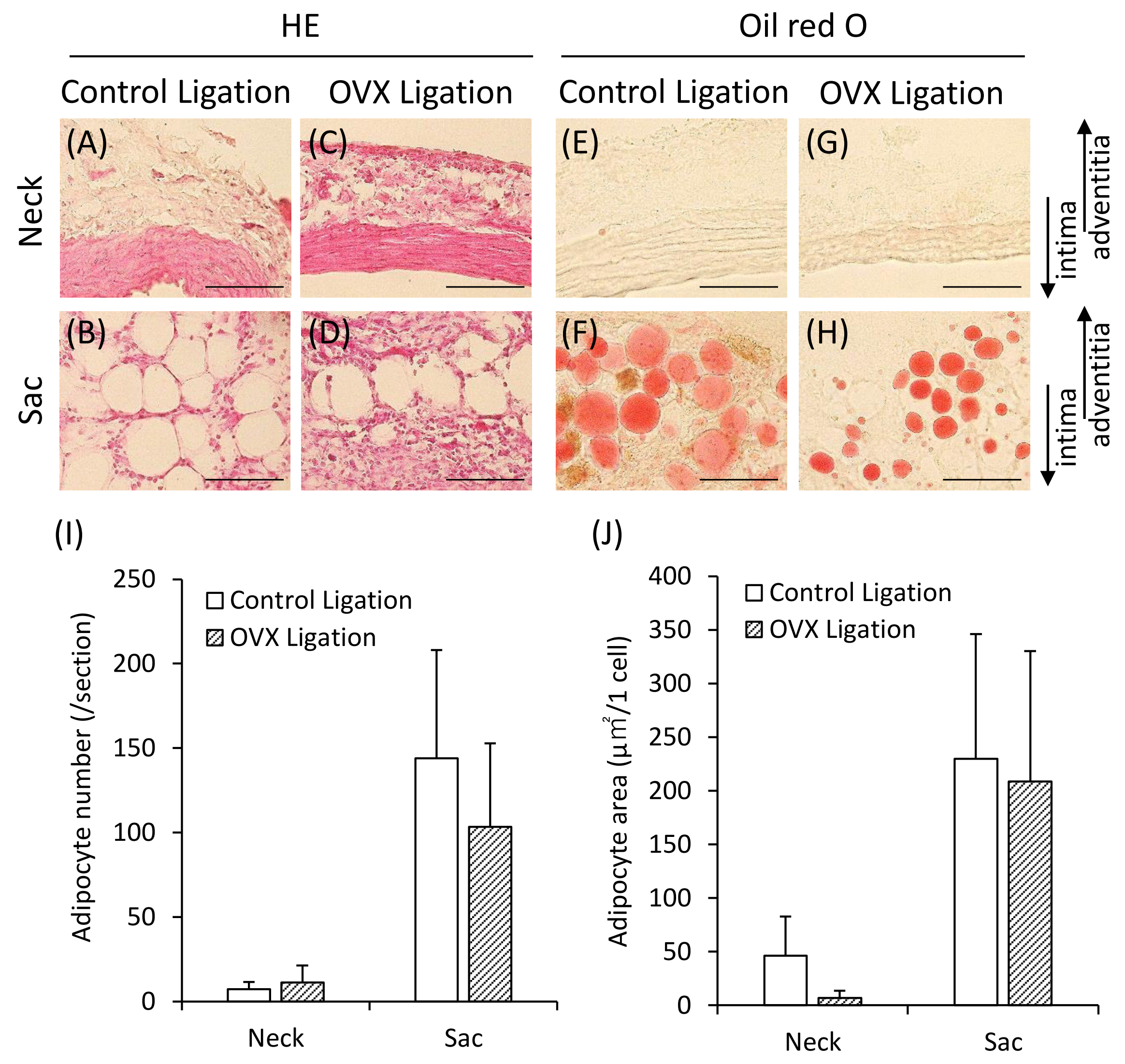

Supplementary Figure S2

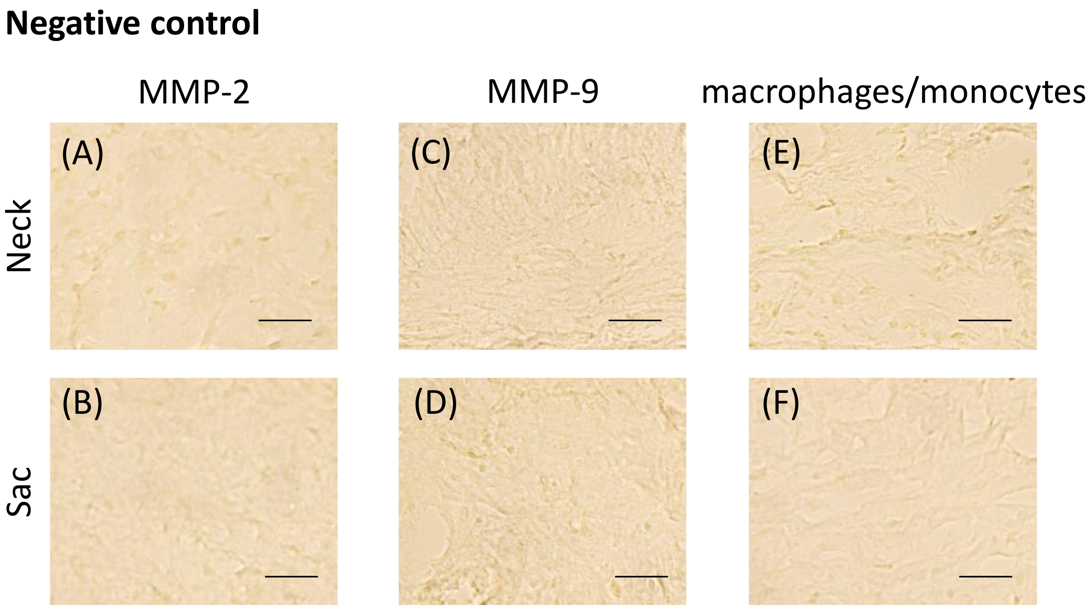

Supplementary Figure S3

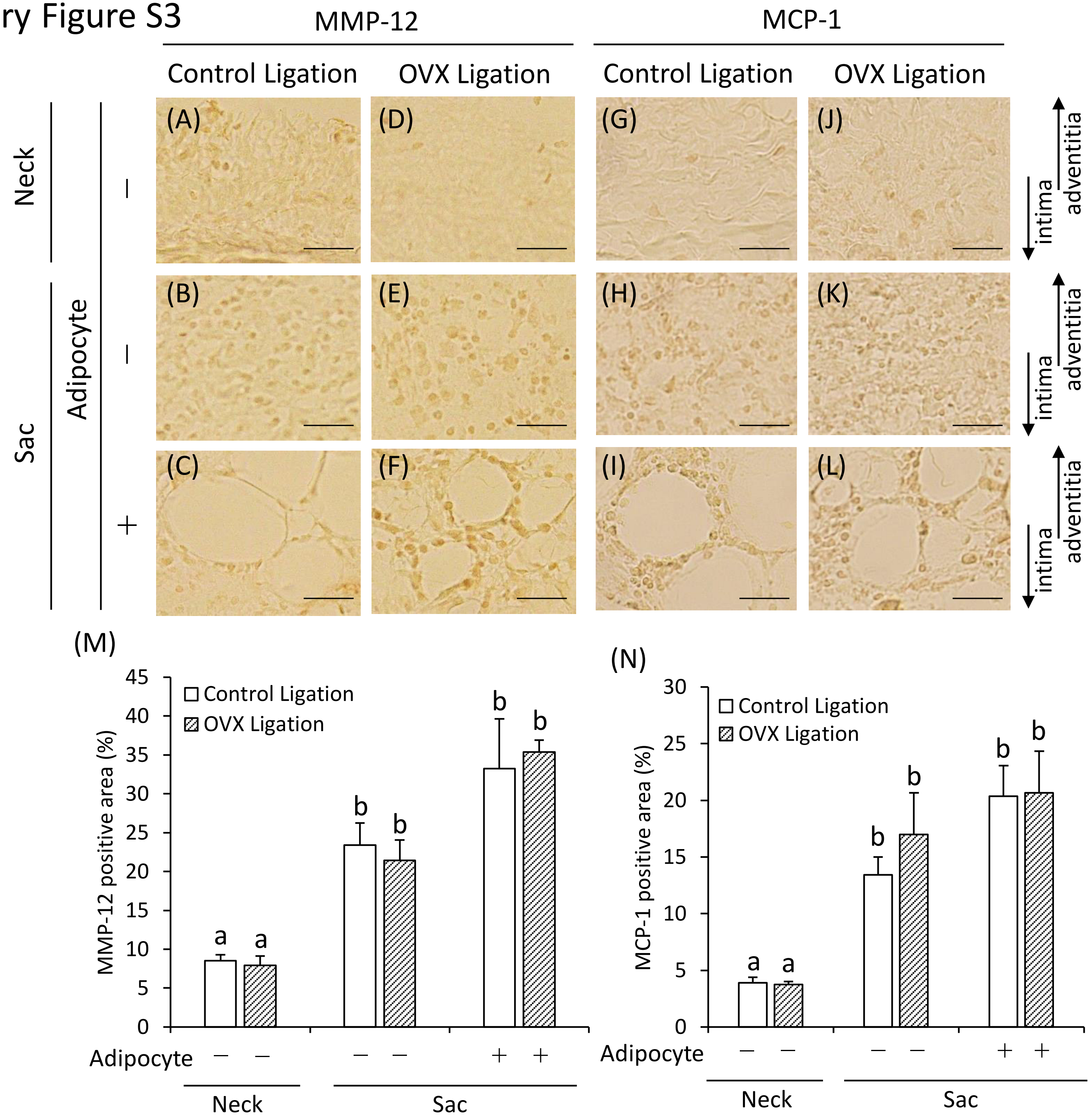

**Supplementary Table S1** Body weight change, food intake, oviduct weight, and serum 17β-estradiol in experimental 1.

| | **Control Ligation** | **OVX Ligation** |
|---|---|---|
| **Initial body weight (g)** | 50.35±1.85 | 47.53±1.85 |
| **Final body weight (g)** | 227.30±18.36 | 241.15±16.00 |
| **Food intake (kcal/day)** | 70.13±0.90 | 69.75±1.25 |
| **Oviduct weight (g/100g body weight)** | 0.271±0.041 | 0.034±0.008** |
| **Serum 17β-estradiol (pg/mL)** | 60.07±1.96 | 39.20±9.00* |

Data are expressed as the mean ± S.E. *p<0.05 or **p<0.01 vs control, Student's t-test.

**Supplementary Table S2** Body weight change, food intake, oviduct weight, and serum 17β-estradiol in experimental 2.

| | **3 weeks after OVX** | | **7 weeks after OVX** | |
|---|---|---|---|---|
| | **Control** | **OVX** | **Control** | **OVX** |
| **Initial body weight (g)** | 46.89±1.17 | 48.71±1.26 | 47.09±1.91 | 45.39±1.70 |
| **Final body weight (g)** | 189.46±5.25 | 201.81±5.77 | 237.11±10.75 | 316.36±6.84*** |
| **Food intake (kcal/day)** | 71.68±1.11 | 74.67±1.69 | 79.75±1.55 | 92.53±1.66*** |
| **Oviduct weight (g/100g body weight)** | 0.209±0.020 | 0.028±0.003*** | 0.224±0.012 | 0.022±0.001*** |
| **Serum 17β-estradiol (pg/mL)** | n.d. | n.d. | 62.97±1.48 | 42.87±7.44* |

Data are expressed as the mean ± S.E. *p<0.05 or ***p<0.001 vs control, Student's t-test. n.d. : not determined.

**Supplementary Table S3** Diet composition.

| | |
|---|---|
| **Choline chloride** | 2 |
| **Cystine** | 3 |
| **AIN-93 vitamin mix** | 10 |
| **Dibasic calcium phosphate** | 13 |
| **AIN-93G mineral** | 32 |
| **Cellulose** | 50 |
| **Sucrose** | 68.8 |
| **Maltodextrin** | 125 |
| **Casein** | 200 |
| **Cornstarch** | 506.2 |
| **Lard** | 20 |
| **Soybean oil** | 25 |
| **Total (g)** | 1055 |
| **kcal/g** | 3.85 |
